# Safety, effectiveness, and cost of long-acting versus intermediate-acting insulin for type 1 diabetes: Protocol for a systematic review and network meta-analysis

**DOI:** 10.1186/2046-4053-2-73

**Published:** 2013-09-10

**Authors:** Andrea C Tricco, Huda M Ashoor, Charlene Soobiah, Brenda Hemmelgarn, David Moher, Brian Hutton, Catherine H Yu, Sumit R Majumdar, Sharon E Straus

**Affiliations:** 1 Li Ka Shing Knowledge Institute, St. Michael’s Hospital, 209 Victoria Street, East Building, Room 716, Toronto, ON M5B 1T8, Canada; 2 Departments of Medicine and Community Health Sciences, University of Calgary, 2500 University Dr NW, Calgary, AB T2N 1N4, Canada; 3 Clinical Epidemiology Program, Centre for Practice-Changing Research, Ottawa Hospital Research Institute, 725 Parkdale Ave, Ottawa, ON K1Y 4E9, Canada; 4 Department of Medicine, University of Alberta, 2J2.00 WC Mackenzie Health Sciences Centre, Edmonton, AB T4V 2R3, Canada; 5 Department of Geriatric Medicine, University of Toronto, 27 Kings College Cir, Toronto, ON M5S 1A1, Canada

## Abstract

**Background:**

Type 1 diabetes mellitus (T1DM) causes progressive destruction of pancreatic beta cells leading to absolute insulin deficiency. Treatment of T1DM requires insulin, and some evidence suggests that longer acting insulin analogues might have a higher effectiveness and greater safety profile compared to intermediate-acting insulin. Our objective is to evaluate the comparative effectiveness, safety, and cost of long-acting insulin versus intermediate-acting insulin through a systematic review and network meta-analysis.

**Design/methods:**

Studies examining long-acting versus intermediate-acting insulin or placebo preparations for adult T1DM patients will be included. The primary outcome is glycosylated hemoglobin (A1C), and secondary outcomes include emergency department and physician visits, hospital admissions, weight gain, quality of life, microvascular complications (e.g., retinopathy), macrovascular complications (e.g., cardiovascular disease), all-cause mortality, incident cancers, and cost. We will include experimental [randomized clinical trials (RCTs), quasi-RCTs, non-RCTs], quasi-experimental (controlled before-after, interrupted time series), observational (cohort), and cost studies, of any duration of follow-up, conducted during all time periods, and disseminated in any language.

We will conduct comprehensive searches of electronic databases from inception onwards, including MEDLINE, Cochrane Central Register of Controlled Trials, and EMBASE. We will also search for difficult to locate and unpublished literature by searching dissertation databases, public health organization websites, and trial registries. After a calibration exercise using our eligibility criteria and data abstraction forms, two reviewers will screen all citations, full-text articles, and abstract data in duplicate. Conflicts will be resolved by team discussion. Using a similar process, the Cochrane Effective Practice and Organization of Care Risk of Bias tool will be used to appraise the risk of bias of experimental and quasi-experimental studies, while the Newcastle Ottawa Scale will be used to assess the methodological quality of cohort studies. If feasible and appropriate, we will conduct a random effects meta-analysis, as well as a network meta-analysis.

**Discussion:**

Our systematic review will be of utility to healthcare providers, policy-makers, T1DM patients and family members regarding treatment options of long-acting versus intermediate-acting insulin preparations.

**Trial registration:**

PROSPERO registry number: CRD42013003610

## Background

Type 1 diabetes mellitus (T1DM) is a chronic condition usually characterized by an autoimmune destruction of pancreatic beta cells, leading to absolute insulin deficiency [1]. T1DM is due to a combination of genetic and environmental factors [1]. The long-term consequences of T1DM can be severe and include microvascular complications, such as retinopathy, neuropathy, and nephropathy, as well as macrovascular complications, including cardiovascular disease, stroke/transient ischemic attack, and peripheral vascular disease [1].

The incidence of T1DM varies geographically, with high rates reported across Europe (4 to 41 per 100,000 people per year) and North America (11 to 25 per 100,000 people per year) [2]. Although T1DM accounts for a small proportion of all diabetes worldwide (range: 5-10%) [1], the incidence of T1DM is increasing [2]. Some estimates suggest a 2.8% increase in the incidence of T1DM per year [2].

Since insulin deficiency occurs in T1DM, the treatment of this condition requires the use of insulin. Basal insulin replacement can be achieved with human or purified porcine intermediate-acting insulin, including isophane insulin (Neutral Protamine Hagedorn; NPH) and zinc insulin (lente) [3] or with long-acting insulin analogues, such as glargine and detemir [4]. Long-acting insulin analogues are more expensive than intermediate-acting insulin [3], yet have a slower absorption and less intra-individual variability of action, which is presumed to improve clinical outcomes [5]. Previous reviews of these agents have found that long-acting insulin analogues significantly reduced glycosylated hemoglobin (A1C) compared to intermediate-acting insulin [4,6,7]. However, none of these reviews included “real-world” evidence from study designs beyond randomized clinical trials (RCTs). For example, evidence from observational studies (e.g., cohort studies) was not included in these reviews. As such, our objective is to evaluate the “real-world” comparative effectiveness, safety, and cost of long-acting insulin versus intermediate-acting insulin in managing T1DM through a systematic review and network meta-analysis.

## Methods/design

We compiled a systematic review protocol and registered it with the PROSPERO database (CRD42013003610). We used the Preferred Reporting Items for Systematic Reviews and Meta-analyses Protocols (PRISMA-P) initiative to guide the reporting of our systematic review protocol [8].

### Eligibility criteria

Experimental studies (RCTs, quasi-RCTs, non-RCTs) and quasi-experimental studies (interrupted time series, con-trolled before and after studies) including adults (aged ≥18 years) with T1DM of any duration who are administered long-acting basal insulin analogue preparations (e.g., glargine, detemir) compared to each other, intermediate-acting insulin (e.g., NPH, lente), or placebo will be included. We will exclude pre-mixed insulin preparations. To examine rare adverse events, we will also include observational (cohort) studies. To examine cost, cost and cost effectiveness studies will be included.

The primary outcome of interest is A1C. Secondary outcomes include emergency department (ED) visits (for hypoglycemia and hyperglycemia), physician visits (for hypoglycemia and hyperglycemia), hospital admissions (for hypoglycemia and hyperglycemia), weight gain, quality of life, microvascular complications (retinopathy, neuropathy, nephropathy), macrovascular complications (cardiovascular disease, stroke/transient ischemic attack, peripheral vascular disease), all-cause mortality, and incident cancers. We will also assess the cost and cost-effectiveness of long-acting basal insulin preparations.

Both published and unpublished material will be included, as well as those disseminated in any language. Studies of all durations of follow-up conducted at any point in time will be included. Our draft eligibility form is presented in Additional file 1 for screening titles and abstracts (or citations) and potentially relevant full-text articles.

### Information sources and literature search

The main information sources are electronic databases, such as MEDLINE, EMBASE, and the Cochrane Central Register of Controlled Trials. This will be supplemented by searching for gray literature, as recommended by the Canadian Agency for Technologies in Health (CADTH) [9]. For example, we will search public health websites (e.g., Public Health Agency of Canada, Health Canada), drug regulatory websites (e.g., Food and Drug Administration; FDA), and clinical trial registries (e.g., World Health Organization International Clinical Trials Search Portal). We will also search authors’ personal files, contact insulin manufacturers, scan the reference lists of included studies and relevant reviews, and contact prolific authors of T1DM long-acting insulin papers.

An experienced librarian will conduct all of the aforementioned literature searches. A draft search strategy for the MEDLINE database (OVID interface) is presented in Additional file 2. Our MEDLINE search strategy will be peer reviewed by another expert librarian using the Peer Review of Electronic Search Strategies (PRESS) checklist [10].

### Study selection process

The draft screening criteria presented in Additional file 1 will be calibrated by conducting a pilot-test using a random sample of 50 citations from the literature search. All team members will screen these citations using the eligibility criteria, and conflicts will be discussed. The eligibility criteria will be revised if deemed necessary by the team or if low agreement is observed (e.g., a kappa statistic ≤ 60%) [11]. Two team members will then screen each citation in duplicate using our online SysRev Tool [12]. Similarly, the draft eligibility criteria will be calibrated for screening potentially relevant full-text articles, which will then be screened by two team members independently. At both the citation level of screening and full-text level of screening, conflicts will be resolved through team discussion.

### Data items and data collection process

Data will be abstracted on the following characteristics at the following levels:

(1) Study-level: study design, year of study conduct, sample size, setting, country of study conduct, type of insulin, dosage of insulin;

(2) Patient-level: type and number of patients, age mean and standard deviation, duration of T1DM, baseline A1C, co-morbidities; and

(3) Outcome level: A1C, ED visits, physician visits, quality of life, cost.

We will abstract outcome results for each of the following points in time: 6, 12, 24 months, and the longest duration of follow-up.

The process for data collection will be similar to the method used for screening. Namely, we will calibrate our data collection form on a random sample of 5–10 included studies. Each team member will collect the data, and the team will meet to discuss conflicts. The data collection form will be revised, as necessary. Subsequently, two team members will conduct all data collection for each study in duplicate. The team has been trained to spot duplicate publications (or companion reports) that use the same group of patients to test a particular intervention. This is particularly an issue when meta-analysis is being considered [13]. Furthermore, our team is trained to contact authors of studies with poorly reported data or for potentially relevant unpublished material identified, for example, through conference abstracts or dissertations.

### Methodological quality/risk of bias appraisal

The methodological quality of cohort studies will be appraised using the Newcastle-Ottawa Scale [14]. The risk of bias of experimental and quasi-experimental studies will be appraised using the Cochrane Effective Practice and Organisation of Care Risk of Bias Tool [15]. Publication bias will be assessed using funnel plots [16]. Finally, studies reporting harms will be appraised using the McHarm tool [17].

### Synthesis of included studies

Firstly, our systematic review results will be reported by describing study characteristics, patient characteristics, and outcome results. We will also describe our literature search results, as well as the methodological quality and risk of bias results using tables, figures, and text.

Secondly, we will evaluate whether we have sufficient data to conduct random-effects meta-analysis [18]. We will ensure that the 95% confidence intervals can be derived using a normal distribution. We will also ensure that the body of literature is sufficiently homogenous in terms of clinical (e.g., patient characteristics), methodological (e.g., study design), and statistical (e.g., forest plot consistency) characteristics. For example, clinicians on the team will use their clinical insight to assess for clinical heterogeneity, methodologists on the team will assess for methodological heterogeneity, and statistical heterogeneity will be calculated using the I^2^ statistical test [19]. If extensive heterogeneity is observed (e.g., I^2^> 75% [19]), we will try to explain this via sub-group analysis and meta-regression analysis [20]. The meta-regression analysis will examine the influence of factors such as baseline A1C values (e.g., < 8% versus ≥ 8%), study size (e.g., < median versus ≥ median), and risk of bias (e.g., high risk of bias versus low risk of bias on the random sequence generation item) on the meta-analysis results. Both meta-analysis and meta-regression analysis will be dependent upon the availability of data. These analyses will be conducted using SAS version 9.2 [21].

To make use of all existing data, we will impute missing measures of variance (e.g., standard deviations, standard errors, 95% confidence intervals). This will be conducted using established methods [22]. To ensure that our imputations do not bias our results, we will conduct a sensitivity analysis, which will entail examining the missing data under both random and nonrandom assumptions [23].

Finally, we will attempt conducting a network (i.e., mixed treatment comparisons) meta-analysis. This analysis is particularly useful when there is a lack of head-to-head studies or when both relevant head-to-head and standard treatment controlled studies exist. The network meta-analysis approach allows the ranking of effectiveness and safety of different insulin preparations [24]. This analysis will be conducted in WinBUGS [25], and median rankings (or point estimates) will be calculated using a random effects model that makes use of all available direct and indirect data [24]. The degree of uncertainty for all point estimates will be reported as 95% credible intervals (CIs), calculated using the 2.5 and 97.5 percentiles obtained via Monte Carlo simulation of 100,000 iterations [24]. We will assess model convergence using trace and history plots, as well as the Gelman Rubin statistic [26]. We will first include RCTs in the network meta-analysis. Subsequently, we will include other experimental studies, quasi-experimental studies, and cohort studies in the network meta-analysis. The consistency of results will be examined by comparing the results obtained via frequentist meta-analysis versus network meta-analysis. This will also be examined statistically, using methods reported elsewhere [27,28].

Our meta-analysis results will be tested for their robustness through sensitivity analyses. For example, we might look at the impact of including/excluding studies with high risk of bias or poor methodological quality, studies with high attrition rates, average adherence between groups, and inclusion of non-RCTs in the analyses. Since the network meta-analysis is dependent on different priors for variance parameters included in the Bayesian approach [24], we will conduct a sensitivity analysis on these characteristics as well.

## Discussion

The incidence of T1DM is increasing annually, and most patients with this chronic condition require the use of insulin. As such, our systematic review results have the potential to influence a large proportion of the population. Our results can be used to inform healthcare providers, policy-makers, T1DM patients, and family members about the comparative effectiveness, safety, and cost of long-acting versus intermediate-acting insulin preparations using real-world evidence.

We will ensure that our results reach our key stakeholders by conducting rigorous and evidence-based knowledge translation strategies. Our review was identified by Canadian policy-makers as a priority topic of the health of Canadians. We developed our protocol in response to their query—a form of integrated knowledge translation. We will ensure that our results are disseminated to these policy-makers through dissemination meetings and policy briefs. We will also translate our results to clinicians and patients by publishing in open access journals, presenting at a conference symposium, and utilizing social media.

## Abbreviations

A1C: Glycosylated hemoglobin; CIs: Credible intervals; ED: Emergency department; FDA: Food and drug administration; NPH: Neutral protamine Hagedorn; PRESS: Peer review of electronic search strategies; PRISMA-P: Preferred reporting items for systematic reviews and meta-analyses protocols; RCTs: Randomized clinical trials; T1DM: Type 1 diabetes mellitus.

## Competing interests

The authors report no conflicts of interests.

## Authors’ contributions

ACT conceived the study, designed the study, helped obtain funding for the study, and helped write the draft protocol. HA registered the protocol with the PROSPERO database and edited the draft protocol. CS edited the draft protocol. BH, DM, BH, CHY, and SRM provided input into the design and draft of the protocol. SES conceived the study, designed the study, obtained the funding, and helped write the draft protocol. All authors read and approved the final protocol.

## Supplementary Material

Additional file 1 Draft eligibility criteria. Click here for file

Additional file 2 Draft MEDLINE literature search. Click here for file
