# Supplementary material for: Safety, effectiveness, and cost of long-acting versus intermediate-acting insulin for type 1 diabetes: Protocol for a systematic review and network meta-analysis
Source: Syst Rev. 2013 Sep 10;2:73. doi: 10.1186/2046-4053-2-73 (PMC3847127; doi:10.1186/2046-4053-2-73)
Supplement: Additional file 1 — Draft eligibility criteria. [file 2046-4053-2-73-S1.doc]

Additional file 1: Draft eligibility criteria

Level 1 screening

1. Does this study include adult patients (aged ≥ 18 years) with type 1 diabetes?

YES____ NO____ UNCLEAR____

1. Is this an experimental, quasi-experimental observational study (cohort), or costing studies?

YES____ NO____ UNCLEAR____

1. Are patients treated with long acting basal insulin analogue preparations?

YES____ NO____ UNCLEAR____

| **Generic name of insulin** | **Trade name(s) of insulin** |
| --- | --- |
| Insulin detemir | Levemir |
| Insulin glargine | Lantus |

1. Does the study compare long acting insulin analogues compared to long acting insulin or intermediate acting insulin preparations or placebo?

YES____ NO____ UNCLEAR____

| **Generic name of insulin** | **Trade name(s) of insulin** |
| --- | --- |
| Isophane insulin (neutral protamine Hagedorn, NPH) | Novolin N, Humulin N |
| Zinc insulin (lente) | Humulin L, Novolin L |
| Insulin detemir (long acting) | Levemir |
| Insulin glargine (long acting) | Lantus |

- If you answer NO to any of these questions, the citation/study will be excluded. All other citations will be included.

Level 2 screening

1. Does this study include adult patients (aged ≥ 18 years) with type 1 diabetes?

YES____ NO____ UNCLEAR____

1. Is this an experimental, quasi-experimental, observational study (cohort), or costing study?

YES____ NO____ UNCLEAR____

1. Are patients treated with long acting basal insulin analogue preparations?

YES____ NO____ UNCLEAR____

| **Generic name of insulin** | **Trade name(s) of insulin** |
| --- | --- |
| Insulin detemir | Levemir |
| Insulin glargine | Lantus |

1. Does the study compare long-acting insulin analogues compared to long-acting insulin or intermediate-acting insulin preparations or placebo?

YES____ NO____ UNCLEAR____

| **Generic name of insulin** | **Trade name(s) of insulin** |
| --- | --- |
| Isophane insulin (neutral protamine Hagedorn, NPH) | Novolin N, Humulin N |
| Zinc insulin (lente) | Humulin L, Novolin L |
| Insulin detemir (long acting) | Levemir |
| Insulin glargine (long acting) | Lantus |

1. Does the study report at least one of the following outcomes?

Glycosylated hemoglobin (A1C); emergency department visits for hypo-/hyperglycemia, physician visits for hypo-/hyperglycemia, hospital admissions for hypo-/hyperglycemia, weight gain, quality of life, microvascular complications (retinopathy, neuropathy, nephropathy), macrovascular complications (cardiovascular disease, stroke/transient ischemic attack, peripheral vascular disease), all-cause mortality, incident cancers, cost,or cost-effectiveness.

YES____ NO____ UNCLEAR____

- If you answer NO to any of these questions, the citation/study will be excluded. All other full-text articles will be included.
