# Supplementary material for: Safety, effectiveness, and cost of long-acting versus intermediate-acting insulin for type 1 diabetes: Protocol for a systematic review and network meta-analysis
Source: Syst Rev. 2013 Sep 10;2:73. doi: 10.1186/2046-4053-2-73 (PMC3847127; doi:10.1186/2046-4053-2-73)
Supplement: Additional file 2 — Draft MEDLINE literature search. [file 2046-4053-2-73-S2.doc]

**Additional file 2: Draft MEDLINE literature search**

1 exp Diabetes Mellitus, Type 1/ [ Type 1 Diabetes Mellitus ]

2 exp Diabetic Ketoacidosis/

3 T1DM.tw,ot.

4 IDDM.tw,ot.

5 (insulin adj depend$).tw,ot.

6 insulin-depend$.tw,ot.

7 insulin?depend$.tw,ot.

8 ("typ$ 1" adj2 diabet$).tw,ot.

9 ("typ$ I" adj2 diabet$).tw,ot.

10 ("typ$ 1" adj2 DM).tw,ot.

11 ("typ$ I" adj2 DM).tw,ot.

12 (acidos$ adj2 diabet$).tw,ot.

13 (juvenil$ adj2 diabet$).tw,ot.

14 (child$ adj2 diabet$).tw,ot.

15 (keto$ adj2 diabet$).tw,ot.

16 (labil$ adj2 diabet$).tw,ot.

17 (britt$ adj2 diabet$).tw,ot.

18 (earl$ adj2 diabet$).tw,ot.

19 (p?ediatric adj2 diabet$).tw,ot.

20 (acidos$ adj2 DM).tw,ot.

21 (juvenil$ adj2 DM).tw,ot.

22 (child$ adj2 DM).tw,ot.

23 (keto$ adj2 DM).tw,ot.

24 (labil$ adj2 DM).tw,ot.

25 (britt$ adj2 DM).tw,ot.

26 (earl$ adj2 DM).tw,ot.

27 (p?ediatric adj2 DM).tw,ot.

28 (auto-immun$ adj2 diabet$).tw,ot.

29 (autoimmun$ adj2 diabet$).tw,ot.

30 ("sudden onset" adj2 diabet$).tw,ot.

31 (auto-immun$ adj2 DM).tw,ot.

32 (autoimmun$ adj2 DM).tw,ot.

33 ("sudden onset" adj2 DM).tw,ot.

34 ("insulin$ defic$" adj2 absolut$).tw,ot.

35 or/1-34

36 Insulin, Long-Acting/ [ long-acting insulin terms ]

37 Insulin/aa [Analogs & Derivatives]

38 Hypoglycemic Agents/

39 (antidiabetic adj agent?).tw,ot.

40 (anti-diabetic adj agent?).tw,ot.

41 (antidiabetic adj drug?).tw,ot.

42 (anti-diabetic adj drug?).tw,ot.

43 antidiabetics.tw,ot.

44 anti-diabetics.tw,ot.

45 (antihyperglyc?emic adj agent?).tw,ot.

46 (anti-hyperglyc?emic adj agent?).tw,ot.

47 antihyperglyc?emics.tw,ot.

48 anti-hyperglyc?emics.tw,ot.

49 (hypoglyc?emic adj drug?).tw,ot.

50 (hypo-glyc?emic adj drug?).tw,ot.

51 (hypoglyc?emic adj effect?).tw,ot.

52 (hypo-glyc?emic adj effect?).tw,ot.

53 hypoglyc?emics.tw,ot.

54 hypo-glyc?emics.tw,ot.

55 (longacting adj2 insulin$).tw,ot.

56 ("long acting" adj2 insulin$).tw,ot.

57 ("longacting" adj2 analog$).tw,ot.

58 ("long acting" adj2 analog$).tw,ot.

59 (semilente adj insulin$).tw,ot.

60 (insulin$ adj2 analog$).tw,ot.

61 (insulin$ adj2 derivativ$).tw,ot.

62 lantus.mp. [ Glargine ]

63 optisulin.mp.

64 Gly$A21.mp.

65 A21Gly$.mp.

66 (gly$ adj1 A21).mp.

67 HOE-901.mp.

68 HOE901.mp.

69 SoloStar.mp.

70 glargin$.mp.

71 glargine.rn. [ CAS Registry Number ]

72 detemir.mp. [ Detemir ]

73 levemir.mp.

74 NN 304.mp.

75 LS-186557.mp.

76 detemir.rn. [ CAS Registry Number ]

77 or/36-76

78 exp Adult/ [ validated geriatric filter - best sensitivity ]

79 adult.mp. [ validated adult filter - best sensitivity ]

80 Middle Aged/

81 age$.tw.

82 or/78-81

83 35 and 77 and 82

84 exp Animals/ not (Humans/ and exp Animals/)

85 83 not 84
